# Pharmacokinetics and Therapeutic Potential of *Teucrium polium* against Liver Damage Associated Hepatotoxicity and Oxidative Injury in Rats: Computational, Biochemical and Histological Studies

**DOI:** 10.3390/life12071092

**Published:** 2022-07-21

**Authors:** Fatma Rahmouni, Riadh Badraoui, Hmed Ben-Nasr, Fevzi Bardakci, Salem Elkahoui, Arif J. Siddiqui, Mohd Saeed, Mejdi Snoussi, Mongi Saoudi, Tarek Rebai

**Affiliations:** 1 Laboratory of Histo-Embryology and Cytogenetics, Medicine Faculty of Sfax, University of Sfax, Sfax 3029, Tunisia; fatmarahmouni1987@gmail.com (F.R.); tarek.rebai@fmsf.rnu.tn (T.R.); 2 Department of Biology, College of Science, University of Ha’il, Ha’il 81451, Saudi Arabia; fbardakci@adu.edu.tr (F.B.); s.elkahoui@uoh.edu.sa (S.E.); ar.siddiqui@uoh.edu.sa (A.J.S.); mo.saeed@uoh.edu.sa (M.S.); m.snoussi@uoh.edu.sa (M.S.); 3 Section of Histology-Cytology, Medicine Faculty of Tunis, Tunis El Manar University, La Rabta, Tunis 1007, Tunisia; 4 Laboratory of Pharmacology, Medicine Faculty of Sfax, University of Sfax, Sfax 3029, Tunisia; hmed.bennasr@fsgf.rnu.tn; 5 Department of Biology, Faculty of Sciences of Gafsa, University of Gafsa, Zarroug, Gafsa 2112, Tunisia; 6 Laboratory of Animal Physiology, Sciences Faculty of Sfax, University of Sfax, Sfax 3064, Tunisia; mongi.saoudi@fss.rnu.tn

**Keywords:** liver steatosis, *Teucrium polium*, CCl_4_, antioxidant, oxidative injury, pharmacokinetics, histopathology, in silico

## Abstract

This study investigated the druggability, pharmacokinetics and ethyl acetate extract of *Teucrium polium* (EA *T. polium*) and the protective effect against carbon tetrachloride (CCl_4_) induced liver cirrhosis in rats. The total antioxidant capacity (TAC) and scavenging activity of the extract were examined. The in vivo protective study was based on the use of an animal model of CCl_4_-induced liver cirrhosis. Four groups of rats have been used: Group I: control rats; Group II: received CCl_4_ in olive oil (0.5 mL/kg); Group III: received the EA *T. polium* (25 mg/kg) of pretreatment for seven days by gavage then CCl_4_ in olive oil by gavage for 15 days. Group IV: received the EA of *T. polium* for seven days (25 mg/kg). EA *T. polium* was found to possess significant antioxidant capacity. CCl_4_ caused a hepatotoxicity associated increase in both levels of AST and ALT, which were reduced back to normal values following EA *T. polium* pretreatment. Hepatotoxicity associated structural modifications of liver tissues and increase in thiobarbituric acid reactive substances (TBARS), conjugated dienes (CD) and carbonyl proteins (CP), associated decreases in several assessed antioxidant enzymes such as superoxide dismutase (SOD), glutathione peroxidase (GPx) and catalase (CAT). The in vivo findings on the protective effect of *T. polium* were supported by its druggability, its pharmacokinetic properties and molecular docking assays. These results confirm the modulatory antioxidant and hepatoprotective potential of *T. polium* in this experimental liver cirrhosis model. *T. polium* phytochemicals are good candidates for further pharmaceutical explorations and drug design.

## 1. Introduction

Oxidative stress plays a crucial role in the progression of liver diseases by toxic xenobiotics [1]. Many drugs derived from plants have been applied for the treatment of such pathologies. Among these, *T. polium* is a plant which belongs to the Labiatae family. Both the structural diversity of natural products and the better understanding of their nutraceutical/biological activities have led to increased interest in their applications in drug discovery and development [2,3,4,5]. *Teucrium* species are commonly used as alimentary plants, and some of them are currently used in herbal teas and infusions of leaves. This genus is very important in food industries because many species are endowed with antioxidant, antimicrobial and antifungal activities which enhance their use as biological preservatives [6,7,8]. It is one of the richest sources of neoclerodane diterpenes, which possess a key structure in several biological activities. Recent records indicate that more than 220 of their secondary metabolites have been described, and many of them showed considerable medicinal properties and/or ecological interest [7,8,9]. It is also commonly used for antibacterial, antispasmodic, antiulcer, anorexic, and antipyretic agents and has a hepato-protective activity against CCl_4_ induced hepato-oxidative stress in rats [10,11]. It has been reported that extracts of *T. polium* are rich in phenolic compounds, which allow them to possess hepato-protection via the inhibition of lipoperoxidation and enhancing the activity of antioxidant enzymes [12]. Recently, using high resolution-liquid chromatography mass spectroscopy (HR-LCMS), it was reported that *T. polium* dominant compounds include several small peptides associated (1) 13R-hydroxy-9E,11Z-octadecadienoic acid, (2) Rhoifolin, (3) Sericetin diacetate, (4) Selinidin, (5) Valtratum, (6) Triptonide, (7) Koparin 2′-Methyl Ether, (8) Dihydrosamidin, (9) 4, 16alpha, 17beta-Estriol 3-(beta-Dglucuronide), (10) Cepharanthine, (11) Carapin-8 (9)-Ene, (12) 1-dodecanoyl-sn-glycerol [7,8]. In previous works by our team, the protective effect of the aqueous extract of *T. polium* against CCl_4_-induced toxicity in rats was assessed. Using ethyl acetate (EA), many more components would be extracted due to the increased polarity and the biological effects might be more efficient. In fact, several studies reported that the use of the plant whole extracts are usually more efficient than the separated compounds.

The current work is devoted to study the protective effect of EA *T. polium* against hepatotoxicity and oxidative stress induced by carbon tetrachloride in rats. Furthermore, the druggability and pharmacokinetic properties have been evaluated for the major *T. polium* phytochemicals based on their absorption, distribution, metabolism, excretion and toxicity (ADMET) attributes. Likewise, the molecular interactions of these phytochemicals with two key receptors; cytochrome P450 (CYP) and the stimulator of interferon genes receptors (STING), which are commonly targeted in the treatment of liver diseases, have been explored.

## 2. Materials and Methods

### 2.1. Preparation of EA T. polium

The aerial parts of the *T. polium* specimens were washed with tap water, dried and then crushed with a mortar. An amount of 100 g of the powder was first subjected to a three-day maceration step in 80% ethanol. The obtained hydroalcoholic filtrate underwent evaporation followed by solubilization in water. Later on, the obtained aqueous phase was subjected to a fractionation step using hexane, butanol and ethyl acetate. The fraction obtained was dried with the lyophilizer and then weighed to calculate the yield. Finally, the resulting fractions were evaporated using a vacuum to get the following weights: 0.79 g for hexane, 1.25 g for butanol and 3.2 g for ethyl acetate. The extracts were stored in the dark at 4 °C until they were used for further analyses.

### 2.2. Total Antioxidant Capacities (TAC) Assay

TAC of EA *T. polium* was measured following the method described by Prieto et al. [13] using the phosphomolybdenum and compared to the ascorbic acid as a standard. The method included the reduction of Mo (VI) to Mo (V) by the assessed specimen, at acidic pH, via the formation of phosphate/Mo (V): a green complex. TAC levels were expressed as mg of ascorbic acid equivalents (AAEs)/gram of dry extract.

### 2.3. Scavenging Activity of ABTS Radical Cation

The trolox equivalent antioxidant capacity (TEAC) method, which is based on the reduction of the 2, 2′-azino-bis (3-ethylbenzothiazoline-6-sulphonic acid) (ABTS) radical cation by the specimen’s antioxidants was used to assess the as previously reported by Laporta et al. [14]. Briefly, the method was assessed spectrophotometrically and the absorbance was measured at 734 nm. Methanol was used as the blank and various concentrations of Trolox (0–20 µM) have been used for the calibration curve. Results were expressed as mM concentration of Trolox/g of dry extract.

### 2.4. In Vivo Antioxidant Properties

#### 2.4.1. Animals Experiments

All the experimental procedure including the animal housing, the way they have been treated, sacrificed… met the international standards (Council of European Communities 2010). This study followed the guidelines for the care and use of laboratory animals at Sciences Faculty of Sfax, University of Sfax (Tunisia), which refers to the National Academies Press (NAP) Guide for the Care and Use of Laboratory Animals (https://www.nap.edu/read/12910/chapter/1 (accessed on 15 October 2021)), and was approved by the local Ethical Committee (12/ES/15-2021).

Male *Wistar* rats weighing of about 180–200 g were purchased from the Central Pharmacy of Tunisia (SIPHAT, Sfax, Tunisia). Standard housing conditions were used during the experimental procedure: 22 ± 3 °C, 12 h/12 h and 40 ± 2%, for average temperature, light/dark periods and humidity, respectively. The rats were given standard food (SICO, Sfax, Tunisia) and drinking water ad libitum. 

#### 2.4.2. Experimental Protocols

For the purposes of this study, 24 rats were divided in four groups of six rats each. Free access was given to both food and water. Dose, duration and route of treatment were selected according to previous published reports in rats and ethno-pharmacological studies [7,8,12].

1. Group I received drinking water for 15 days and constituted the control group.
2. Group II received 0.5 mL/kg of CCl_4_ in olive oil by gavage for 15 days and constituted the CCl_4_ group.
3. Group III received a seven-day pretreatment with the EA *T. polium* (25 mg/kg) then received 0.5 mL/kg of CCl_4_ in olive oil for 15 days. Both EA *T. polium* and CCl_4_ were given by oral gavage. This group constituted the CCl_4_ + EA *T. polium* group.
4. Group IV received the EA of *T. polium* (25 mg/kg) by gavage for 15 days and constituted the EA of *T. polium* group.

#### 2.4.3. Samples Collection and Preparation

At the end of the experimental period, all rats were sacrificed by decapitation. Serum samples were collected following blood centrifugation (4000 rpm, 15 min, 4 °C). The organ samples were also collected from each rat and used for biochemical and histological studies. 

#### 2.4.4. Serum Parameters

Standard commercial kits referenced as 80127 and 92027 (France) were used to measure the serum activity of alanine aminotransferase (ALT) and aspartate aminotransferase (AST), respectively. The activity of these enzymes was expressed as IU/L.

#### 2.4.5. Thiobarbituric Acid Reactive Substances (TBARS) Measurement

Measurement of TBARS as estimation of the lipid peroxidation was determined spectrophotometrically according to the procedure of Yagi [15]. The absorbance was read at 530 nm and TBARS levels were expressed as nmol/mg protein.

#### 2.4.6. Determination of Carbonyl Protein Content

Levels of carbonyl protein were recorded using the method of Ardestani et al. [16]. Briefly, 1 mL of 2, 4-dini-trophenylhydrazine (DNPH, 10 mM) in 2M HCl was mixed with 1 mg of liver samples, then incubated at room temperature for 30 min. Cold TCA (1 mL of 10%, *w/v*) was added and the mixture was centrifuged (3000× *g* for 10 min). Ethanol/ethyl acetate (2 mL, 1:1, *v/v*) was used to wash the protein pellet, which was finally dissolved in of guanidine hydrochloride (1 mL, 6 M, pH 2.3). The absorbance was checked at 370 nm and the carbonyl contents were expressed as nmol/mg protein.

#### 2.4.7. Assessment of Conjugated Dienes (CDs)

CDs were assessed as previously described by Slater (1984) [17] based on the use of a chloroform-methanol mixture (2:1) and centrifugation at 1000× *g* for 5 min. After evaporating the chloroform at 50 °C, the resulted lipid residue was dissolved in methanol (1.5 mL) and the absorbance was recorded at 233 nm.

#### 2.4.8. Superoxide Dismutase (SOD) Assay

The SOD levels were measured as described by Asada et al. [18], which is based on the inhibition of nitro-blue tetrazolium (NBT). Briefly, 1 mL of the liver homogenate was mixed with phosphate buffer (50 mM, pH 7.8), methionine (39 mM), NBT (2.6 mM) and EDTA-Riboflavin (2.7 mM). Twenty minutes after starting the reaction by turning on the light, the changes in the absorbance were followed at 560 nm. One unit of SOD corresponded to the required amount to inhibit 50% of NBT photoreduction. SOD activity is reported as Units/mg of protein.

#### 2.4.9. Glutathione Peroxidase (GPx) Assay

GPx activity was assessed spectrophotometrically as reported by Flohe and Gunzler [19]. The liver supernatant issued by centrifugation was mixed with phosphate buffer (0.1 M pH 7.8) and DTNB (0.4 mg/mL) at 0.2 and 0.7 mL, respectively. The absorbance was recorded at 420 nm. GPx activity was expressed as µmol GSH min^−1^ mg protein^−1^.

#### 2.4.10. Catalase (CAT) Assay

CAT was assessed using the method previously described by Aebi [20]. The reaction started by adding H_2_O_2_ to the reaction mixture. The decrease in the absorbance, as decomposition of H_2_O_2_ was recorded for 1 min, at 420 nm. CAT activity was expressed as µmol H_2_O_2/_mg protein.

#### 2.4.11. Protein Measurement

Protein measurement in liver samples was studied using the method of Lowry et al. (1951), in which bovine serum albumin was used as a standard.

### 2.5. In Silico Molecular Interactions and Pharmacokinetic Assays

**Figure 1:**
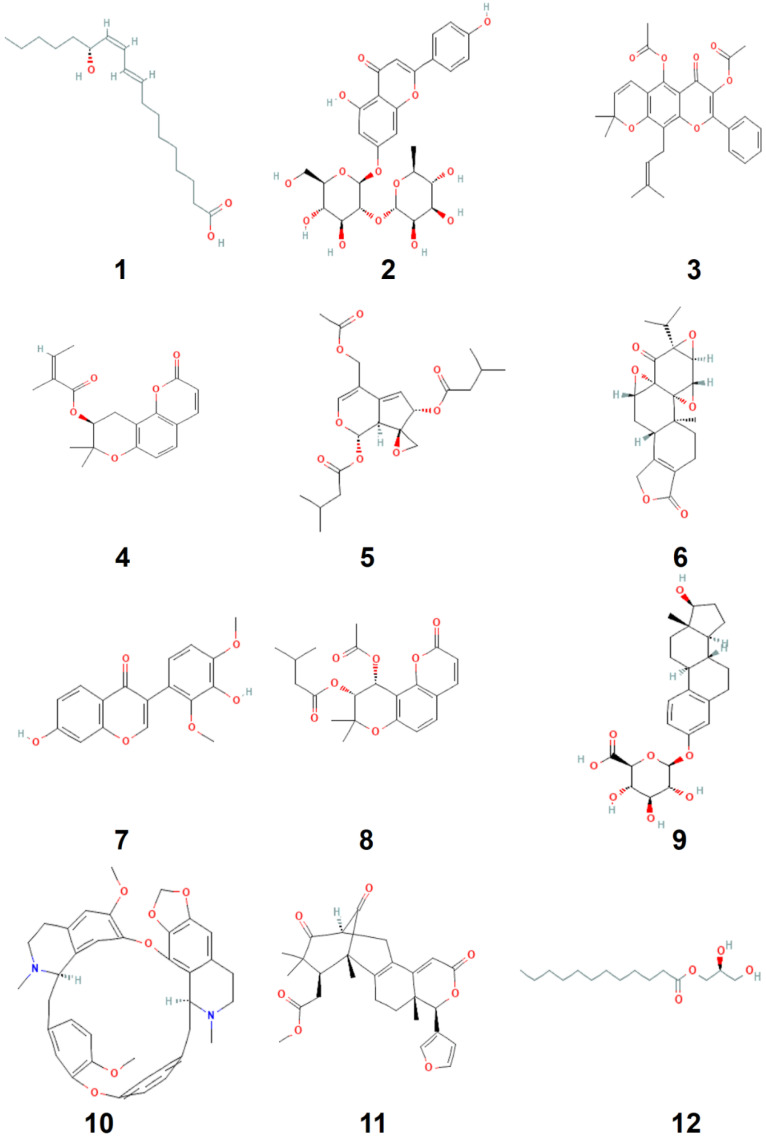
Chemical structure of the *T. polium* identified compounds. (1) 13R-hydroxy-9E,11Z-octadecadienoic acid, (2) Rhoifolin, (3) Sericetin diacetate, (4) Selinidin, (5) Valtratum, (6) Triptonide, (7) Koparin 2′-Methyl Ether, (8) Dihydrosamidin, (9) 4, 16alpha, 17beta-Estriol 3-(beta-Dglucuronide), (10) Cepharanthine, (11) Carapin-8 (9)-Ene, (12) 1-dodecanoyl-sn-glycerol.

The physicochemical and pharmacokinetic properties of the *T. polium* phytochemicals, which we had recently determined [7,8], were assessed. ChemDraw Pro 12.0 was used to draw the chemical structures of these phytochemicals (Figure 1). The most pertinent physicochemical parameters were used to assess the draggability and pharmacokinetic properties based on ADMET. These parameters have been assessed in March 2022 using ADMET properties. The tridimensional (3D) structure of CYP and STING receptors (1OG2 and 6DXL respectively), (Doi: 10.2210/pdb1OG2/pdb and 10.2210/pdb6DXL/pdb respectively), and selinidin, (compound no. 4, Accession number: 668079) were obtained from the RCSB PDB and pubchem^®^, respectively. Both ligand and receptors were processed for minimization and saved in pdbqt format as previously described [21,22,23].

The binding assessment used in molecular docking and calculation of the binding energy was based on the CHARMm force field using MGL tools and Vina^®^ software (version 4.2.6) as previously described [3,21,24]. The ability of the ligands (major component of *T. polium*: selinidin) to bind to androgen receptors CYP-LBD and STING-LBD was studied after the removal of heteroatoms (warfarin) and water molecules and adding Coleman charges. Both CYP and STING receptors are involved in xenobiotic biotransformation and the etiology of diverse liver pathology including hepatotoxicity, liver cancer and infectious diseases [25,26].

### 2.6. Histopathological Analysis

The liver was collected and fixed in 10% formalin and then processed for standard histological preparation. Several sections of 5 µm-thick were prepared and then stained with hematoxylin and eosin (H&E). The histological slides have been examined for histopathological changes under an optic microscope.

### 2.7. Statistical Analysis

All results were expressed as mean ± standard deviation. To check the significant differences between the different groups, a one-way ANOVA and Tukey’s post hoc test were carried out using the SPSS software package. Differences were considered significant whenever *p* < 0.05.

## 3. Results

Extraction with different solvents showed that EA *T. polium* has a better activity compared with those of two other solvents; hexane and butanol. 

### 3.1. TAC of EA T. polium In Vitro

The TAC of EA *T. polium* was measured spectrophotometrically using the phosphomolybdenum method. The TAC of EA *T. polium* extract was 123.85 ± 60 mg AAEs/g of dry extract (Table 1). In this study, the ABTS radical scavenging assay was also used. The principle of the method is based on the ability of an antioxidant to stabilize the ABTS^+^ by trapping a proton with the antioxidant. A comparison was made with the ability of Trolox to capture ABTS^+^. Our results showed that *T. polium* has a significant antioxidant capacity equal to 0.62 mM equivalents Trolox/mg of extract (Table 1). A statistical analysis showed that both TAC and ABTS scavenging activity of EA *T. polium* were much better than hexane and butanol extract.

### 3.2. Serum Biochemical Parameters

The activities of AST and ALT, in the different studied groups, are shown in Table 2. Both AST and ALT activities increased significantly in the CCl_4_ group once compared to control. Pretreatment with EA *T. polium* decreased the activity of the above enzymes with statistically significant levels. Animals given EA *T. polium* showed no disrupted levels of ALT and AST as compared to the control group.

### 3.3. Oxidative/Antioxidative Status

The administration of CCl_4_ significantly elevated the TBARS level, an indicator of lipid peroxidation, while the pretreatment with EA *T. polium* resulted in the reduction of the serum activity of liver marker enzymes when compared to CCl_4_ intoxicated rats. This hepatoprotective activity may be due to the anti-oxidant activity of EA *T. polium*. The effects of CCl4, EA of *T.polium* and their combination on serum conjugated dienes (CD) and carbonyl protein (CP) in the different groups are shown in Table 3. As compared to controls, the levels of these parameters were significantly increased in CCl_4_ treated rats. The pretreatment with EA *T. polium* lowered the level of these parameters. No difference was detected between the group pretreated with EA *T. polium* and control.

The lipid peroxidation was associated with a concomitant decline in the activity of antioxidant enzymes such as SOD, GPx and CAT (enzymes involved in the defense against oxidative stress) respectively in the CCl_4_ treated rats compared to the control rats. However, pretreatment with EA *T. polium* significantly restored the activity of antioxidant enzymes in CCl_4_ treated rats (Table 4). The administration of EA *T. polium* was also able to significantly restore the enzyme level compared to CCl_4_ alone.

### 3.4. Druggability and Pharmacokinetic Properties of EA T. polium Main Compounds

**Figure 2:**
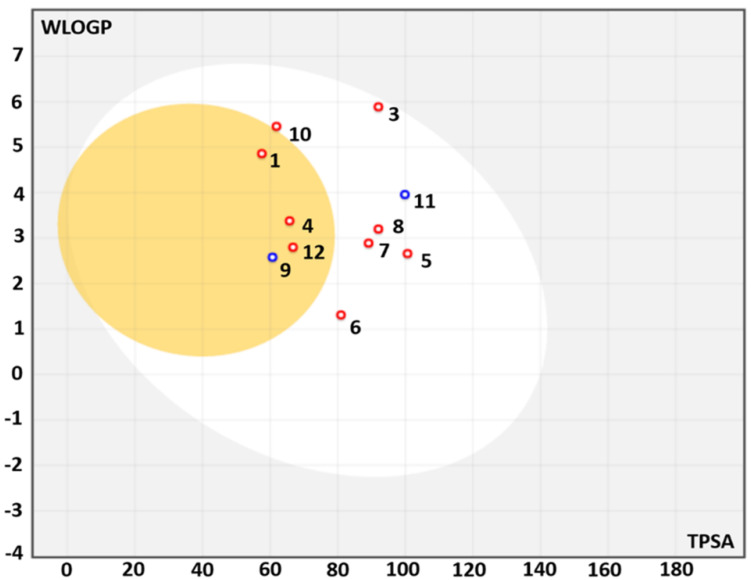
Boiled-egg model of the *T. polium* studied compounds using ADMET properties. (1) 13R-hydroxy-9E,11Z-octadecadienoic acid, (2) Rhoifolin, (3) Sericetin diacetate, (4) Selinidin, (5) Valtratum, (6) Triptonide, (7) Koparin 2′-Methyl Ether, (8) Dihydrosa-midin, (9) 4, 16alpha, 17beta-Estriol 3-(beta-Dglucuronide), (10) Cepharanthine, (11) Carapin-8 (9)-Ene, (12) 1-dodecanoyl-sn-glycerol.

**Figure 3:**
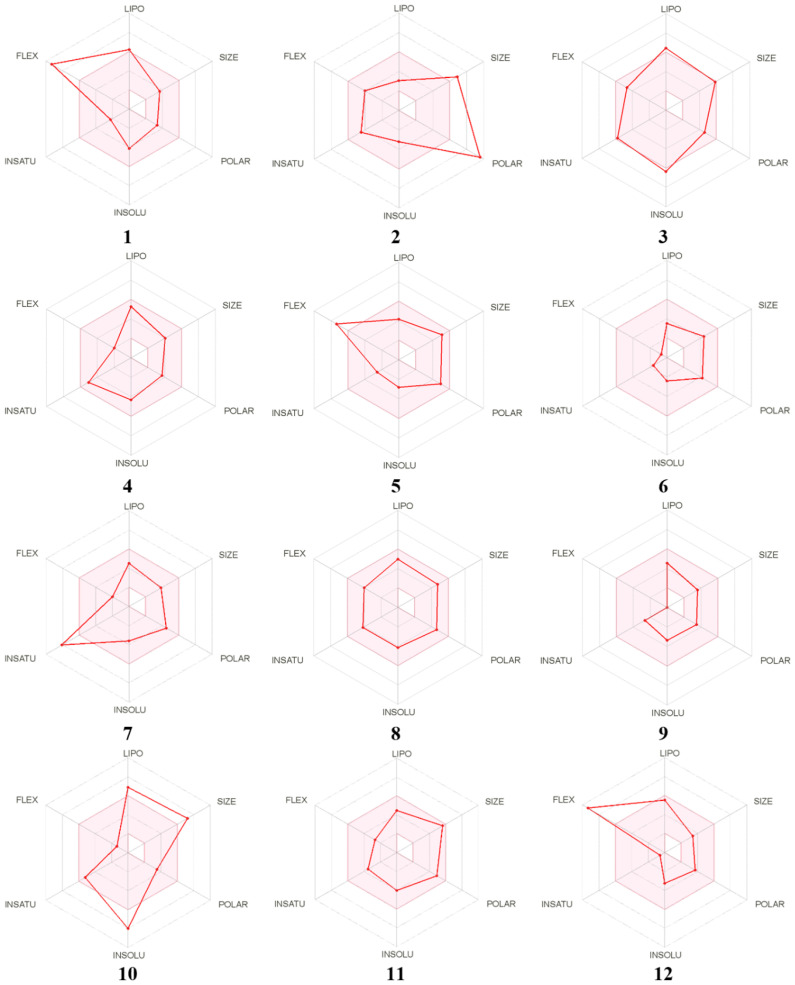
Bioavailability polygons of *T. polium* identified compounds based on their physicochemical parameters using ADMET properties. (1) 13R-hydroxy-9E,11Z-octadecadienoic acid, (2) Rhoifolin, (3) Sericetin diacetate, (4) Selinidin, (5) Valtratum, (6) Triptonide, (7) Koparin 2′-Methyl Ether, (8) Dihydrosa-midin, (9) 4, 16alpha, 17beta-Estriol 3-(beta-Dglucuronide), (10) Cepharanthine, (11) Carapin-8 (9)-Ene, (12) 1-dodecanoyl-sn-glycerol.LIPO: Lipophilicity, SIZE: Molecular size, POLAR: Polarity, INSOLU: Insolubility, INSATU: Insaturation, FLEX: Flexibility, The pink area represents the most suitable physicochemical zone for oral bioavailability. Note that some compounds, including selinidin, stand in the pink area, which is the most suitable for oral bioavailability.

The previous *T. polium* identified compounds were studied to assess their druggability and pharmacokinetic properties [7,8]. Interestingly, the studied compounds displayed nil alert. Furthermore, regardless of the compound no. 2 (rhoifolin), which stands out of the boiled egg model (Figure 2), all the other phytochemical compounds had acceptable consensus Log *P*_o/w_, obeyed to the Lipinski’s rule and have good gastro-intestinal absorption, lipophilicity and bioavailability score (0.55–0.58). This permit these compounds to be placed in the pink zone of the bioavailability polygons (Figure 3). Moreover, all the assessed *T. polium* compounds are not suitable for transdermal delivery. All the *T. polium* identified phytochemicals displayed negative skin permeability ranging from −0.55 to −9.94 cm/s (Table 5), which indicates low to moderate skin permeability. These results might support the selected route of *T. polium* administration in our study (gavage) and their potential biological activities: antioxidant and hepato-protective effects. Selinidin, which possessed good pharmacokinetic properties and oral bioavailability, have been further assessed for potential hepato-protective effect using in silico analyses.

### 3.5. In Silico Molecular Interactions

**Figure 4:**
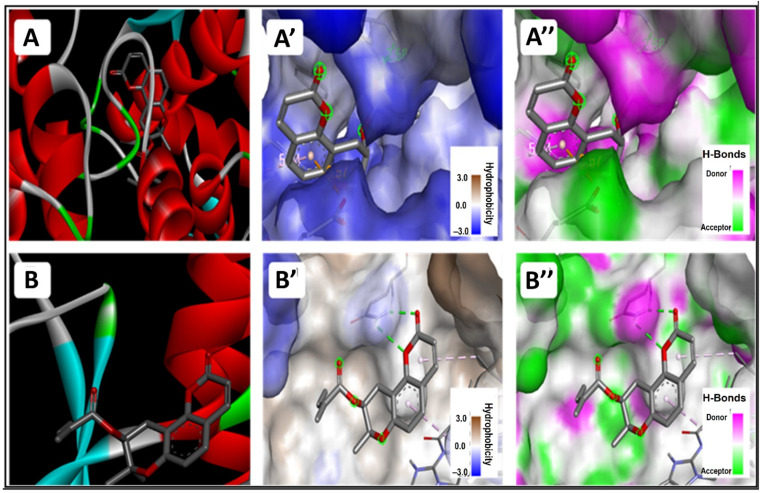
Photograph of the 3D structure of selinidin bounded to the ribbon structure of cytochrome P450 (top: **A**) and stimulator of interferon genes (STING, bottom: **B**) receptors. 3D interaction’s illustrations of the hydrophobicity (**A’** and **B’** respectively) and conventional hydrogen bond (**A’’** and **B’’** respectively).

**Figure 5:**
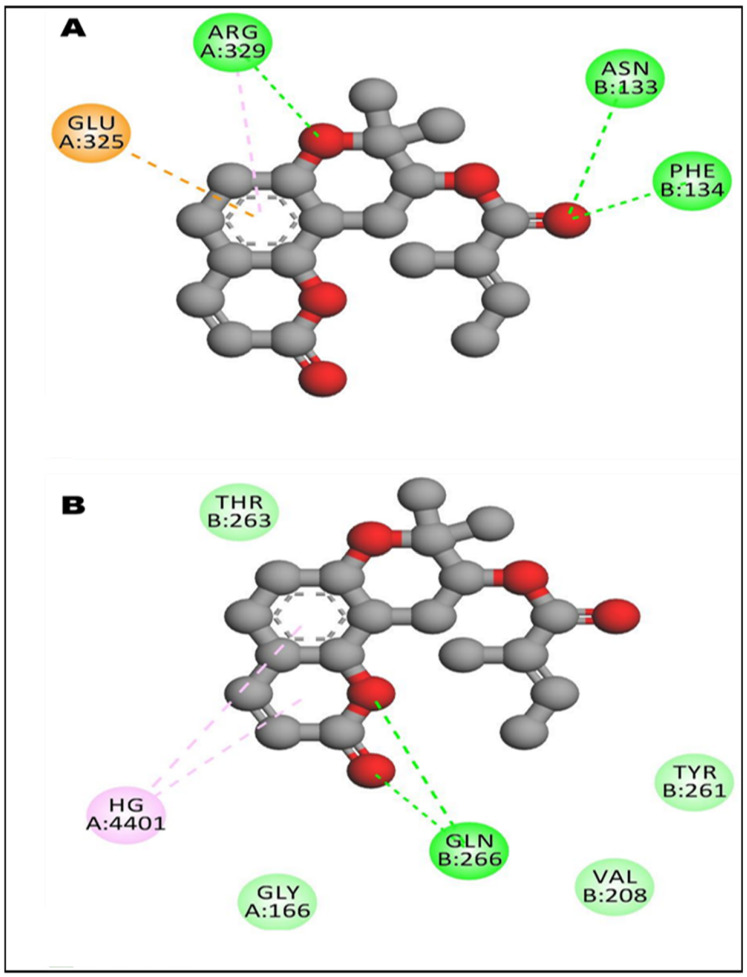
Illustration of the 2D diagrams of interactions with the pocket region of CYP (**A**) and STING (**B**) receptors, respectively.

Selinidin established three H-bonds with CYP via both its heterocyclic ring (with Arg329) and its branched chain (with Asn133 and Phe134). With STING receptor, the Gln266 established a couple of H-bonds with the first heterocyclic ring (Figure 4 and Figure 5). Furthermore, selinidin was found to be strongly embedded particularly with the pocket region of the CYP receptor. It showed a network of conventional H-bonds, electrostatic and hydrophobic bonds. 

### 3.6. Histopathological Study

**Figure 6:**
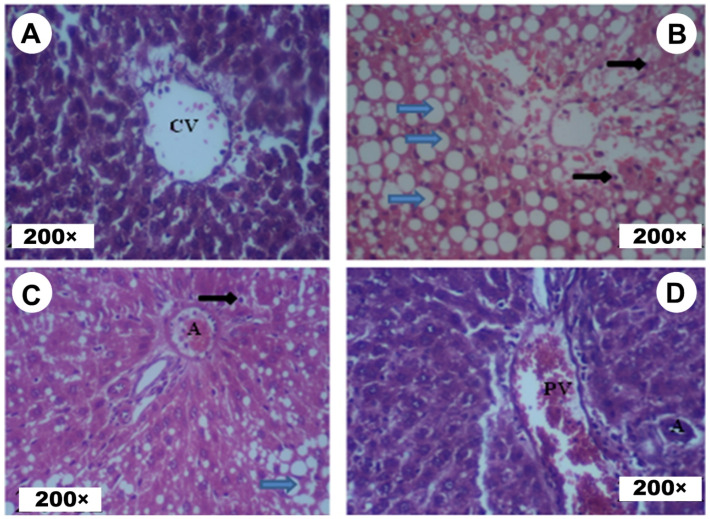
Histological study of the liver: (**A**) = Control group; (**B**) = CCl_4_ group; (**C**) = CCl_4_ + EA *T. polium* group and (**D**) = EA *T. polium* group. CV: Centrilobular Vein; Blue arrows: Hydropic Degenerations; Black arrows: Hepatic Necrosis; A: Arteriole; PV: Portal Vein.

Histological study performed on the liver showed healthy liver parenchyma (normal liver cells, centrilobular and sinusoidal veins and normal hepatocyte trabeculae) in the control rats. Moreover, there was a severe alteration of the architecture with the presence of inflammatory aspects, leukocyte infiltration and cytoplasmic vacuolization of the hepatocytes and hepatic necrosis in CCl_4_ treated rats. These modifications were less pronounced in CCl_4_ + EA *T. polium* treated rats. This study shows that there was conservation of the usual liver structure in rats treated with EA *T. polium* in comparison to controls (Figure 6). These results parallel both biochemical and oxidative/antioxidative status findings.

## 4. Discussion

Antioxidants from medicinal herbs may play an important protective role against oxidative injury and maintain the healthy physiological function of cells. *T. polium* was reported to possess a potent scavenging activity against the reactive oxygen species [7,8,11,27]. Hence, the obtained results data revealed that the EA fraction of the studied plant possesses potent anti-oxidant properties. The results of our study supported those of Movahedi et al. [28] who demonstrated that the EA extract of *T. polium* possessed the highest antioxidant activity by DPPH and FRAP tests, and by total phenolic and flavonoid contents. Our results are also in agreement with previous research activities which revealed that *T. polium* possessed an antioxidant activity by DPPH method and the IC_50_ values were about 90 μg/mL [7,8,29]. These results parallel both the biochemical and oxidative/antioxidative findings.

AST and ALT enzymatic activities associated carbohydrate and amino acid metabolisms. The increased activity of these enzymes reflects liver injury and the inflammation of hepatic parenchyma [1]. Our results revealed that that of CCl_4_ induced significant increases in both AST and ALT activities, which corroborate the findings of previous studies that also reported the interest of phytotherapy in reducing the increases of these parameters comparatively with CCl_4_ treated rats [30].

Our results supported those of Rahmouni et al. [31], who showed that subchronic exposure to CCl_4_ increased TBARS levels and decreased antioxidant enzymes. Our studies also supported those of Sun et al. [32], who reported new insights into the acute liver pathogenesis via CCl4 and some potential novel strategies for its treatment. Similarly, Bellassoued et al. (2018) evaluated the protective effect of *Mentha piperita* L. (which also belongs to Lamiacee family), and reported potential benefits mainly due to menthol and its derivatives.

A recent phytochemical screening study using HR-LCMS revealed that the *T. polium* composition included several beneficial compounds and small peptides [7,8]. The phytochemical profiling of the plant was associated with promising bioactivities such as antioxidant, anticancer, antibacterial and antiviral effects [7,8]. In this study, these compounds were assessed for their draggability and pharmacokinetics based on their ADMET characteristics, as previously described [5,22,33]. The assessment of such parameters was reported to be important to avoid drug failure in the advanced steps of clinical applications [23,24]. The majority of the compounds obeyed the Lipinski’s rule and all of them were associated with zero alerts as they meet the Lipinski rule of five. Such results indicate the safe use of the *T. polium* phytochemicals and/or extracts as reported in previous studies, [7,8] and supported its ethno-pharmacological applications [8,31]. The partition coefficient between octanol and water, as an indicator of lipophilicity (Log *P*_o/w_), also indicated that all the assessed compounds, except no. 2, possessed acceptable lipophilicity, as Log *P*_o/w_ varied between 1.93 and 5.36. Previous reports indicated that Log *P*_o/w_ < 5 is usually associated with good lipophilicity [5,21,22,33]. Their bioavailability scores prove their potential biological activities and the possibility of oral administration. This was further confirmed by the bioavailability polygons (Figure 3). Regardless of compound No. 2, all *T. polium* studied phytochemicals possessed high gastro-intestinal absorption (GI). Compounds No. 1, 9 and 12 were categorized as blood-brain-barrier (BBB) permeant. The majority of *T. polium* identified compounds were predicted not to be a substrate of P-glycoprotein (P-gp), which indicates the absence of significant disruption of drug transport [21,22]. Compounds No. 2, 4, 6, 10 and 11 inhibited none of the targeted CYP isoforms CYP1A2, CYP2C9, CYP2C19, CYP2D6, and CYP3A4. None of the remaining compounds was predicted to be a potent inhibitor of the CYP studied isoforms, thus, indicating the lack of disrupted metabolism and excretion [21,23]. Log Kp varied between –0.55 and –9.94, which indicated moderate to low skin permeability. Taken together, *T. polium* sounds containing suitable drug-like molecules with good synthetic scores that might have promising systemic effects, including alleviation of hepatic disorders.

CYP and STING receptors have been selected, as they are commonly associated with both liver pathogenesis and are targeted in drug design and development [34,35]. The molecular interactions of *T. polium*’s major compounds with these key receptors, which are targeted in liver therapy, were studied. Both CYP and STING are considered key areas of focus for liver diseases, and thus constituted excellent targets for liver medication [36,37].

The selinidin heterocyclic ring and branched chain were predicted to establish three H-bonds with CYP residues: Arg329, Asn133 and Phe134. With STING receptor, the Gln266 established a couple of H-bonds with the first selinidin heterocyclic ring. Moreover, selinidin was found to be strongly embedded, particularly with the pocket region of the CYP receptor. In fact, the distance to the closest residues was 1.811 Å for CYP and 2.461 Å for STING receptors. These results prove that a hepatoprotective effect of the studied extract is possible. The in silico and molecular docking results of the current study mirror the pharmacokinetic, biochemical and histological findings. Furthermore, selinidin associated with the other phytochemical compounds of *T. polium* may possess a synergistic effect. These molecular interactions may justify the in vivo findings in rats, specifically the hepato-protective potential of the *T. polium* extract via its rich phytochemical profile. It was previously suggested that the use of the cure plant extracts normally has better effects than the plant phytochemical components [3,24,38,39]. In this study, the whole extract was used, and further analyses of molecular dynamics may support our results.

Similar results were obtained by Sakr et al. in rats intoxicated by CCl_4_ [40]. In fact, the liver is a major metabolizing organ for which the biochemical markers are commonly disturbed by toxic products including CCl_4_ [1,41,42]. The current results parallel those of Sreelatha and Padma [43] and Seghatoleslam et al. [44], who reported liver damage associated severe pathological/degenerative features following subchronic exposure to CCl_4._ This study proved the conservation of the usual liver structure in rats treated with EA *T. polium* in comparison to controls.

## 5. Conclusions

Our study revealed that *T. polium* displayed an efficient antioxidant activity using both in vitro and in vivo tests such as ABTS scavenging, TAC, TBARS, SOD... EA *T. polium* was proven to be effective in preventing CCl_4_ induced hepatic injury in rats by restoring both the levels of liver biochemical markers (AST and ALT) and oxidative/antioxidative status, which resulted in correction of liver histological morphology. Druggability, pharmacokinetics and molecular docking assays support the in vitro and in vivo approaches. In fact, the in silico results suggest the satisfactory draggability and the promising properties of the *T. polium* phytochemicals, particularly selinidin. The therapeutic potential may include CYP and STING receptors as assessed by docking and molecular interactions. Further investigations regarding the molecular dynamics of *T. polium* components with several receptors are needed for a better understanding of the pathway and the action mechanism to encourage the use of the plant extract against liver injury. In fact, its beneficial effects are promising and encouraging for its use in pharmaceutical processes.

## Figures and Tables

**Table 1:** Antioxidant activity of EA *T. polium* by TAC and ABTS tests.

Parameters/Fractions	Hexane	Butanol	Ethyl Acetate
**TAC** (EAAs/g extract)	60.21 ± 33.02	92.11 ± 36.33	123.85 ± 60 *
**ABTS** (mM Equivalents Trolox/mg extract)	0.11 ± 0.02	0.23 ± 0.06 *	0.62 ± 0.14 ***^###^

Values are mean ± SD of three distinct measurements. * *p* < 0.5 vs. Hexane. *** *p* < 0.001 vs. Hexane. ^###^ *p* < 0.5 vs. Butanol.

**Table 2:** Effects of CCl_4_, EA of *T. polium* (25 mg/kg) and their combination on serum ALT and AST activity (IU/L).

Enzymes (IU/L)	Control	EA *T. polium*	CCl_4_	CCl_4_ + EA *T. polium*
**ALT**	1738 ± 44.3 ^##^	1768 ± 73.74 ^#^	1913 ± 37.08 **^##^	1805 ± 29.39
**AST**	137.7 ± 16.47	139.8 ± 5.48	575.5 ± 58.72 ^##^	523.5 ± 52.42 ^##^

Values are mean ± SD for six determinations. ** *p* < 0.01 vs. Control. ^#^ *p* < 0.5 vs. CCl_4_. ^##^ *p* < 0.01 vs. CCl_4_.

**Table 3:** Thiobarbituric acid reactive substances (TBARS), carbonyl protein (CP) and conjugated diene (CD) levels of control and treated rats with CCl_4_, EA of *T. polium* (25 mg/kg) and their combinations (CCl_4_ + EA *T. polium*).

Treatment/Parameters	Control	EA *T. polium*	CCl_4_	CCl_4_ + EA *T. polium*
**TBARS** (µmol/mg protein)	5.96 ± 3.09	5.90 ± 2.97	10.2 ± 3.53 **	8.2 ± 2.80 **^##^
**Carbonyl protein** (nmol/mg protein)	0.69 ± 0.023	0.69 ± 0.007	1.38 ± 0.013 ***	1.296 ± 0.004
**Conjugated dienes** (nmol/mg protein)	9 ± 0.02	9.45 ± 0.14	13.21 ± 0.04 **	11.11 ± 0.37 ^#^

Values are mean ± SD for six determinations. ** *p* ≤ 0.01 and *** *p* ≤ 0.001 vs. Control group. ^#^*p*< 0.05 vs. CCl_4_ group. ^##^ *p*< 0.01 vs. CCl_4_ group.

**Table 4:** Effects of CCl_4_, EA *T. polium* (25 mg/kg) and their combination (CCl_4_ + EA *T. polium*) on the SOD, GPx and CAT enzymatic activities in hepatic tissues of different studied groups.

Parameter/Treatment	Control	EA *T. polium*	CCl_4_	CCl_4_ + EA *T. polium*
**SOD** (Units/mg protein)	1.62 ± 0.19	1.65 ± 0.27	0.44 ± 0.09 ***	0.51 ± 0.01
**GPx** (µmol GSH min^−1^ mg protein^−1^)	0.91 ± 0.014	0.92 ± 2.5	0.49 ± 0.01 ***	0.93 ± 0.01 ^#^
**CAT** (µmol H_2_O_2_/mg protein)	174.02 ± 44.3	176.32 ± 22.18	85.9 ± 6.25 ***	149.4 ± 44.29 ^##^

Values are mean ± SD for six rats in each group. *** *p* ≤ 0.001 vs. Control group. ^#^ *p*< 0.05, ^##^ *p*< 0.01 vs. CCl_4_ group.

**Table 5:** Draggability and pharmacokinetic properties of *T. polium* main compounds based on the ADMET properties. (1) 13R-hydroxy-9E,11Z-octadecadienoic acid, (2) Rhoifolin, (3) Sericetin diacetate, (4) Selinidin, (5) Valtratum, (6) Triptonide, (7) Koparin 2′-Methyl Ether, (8) Dihydrosa-midin, (9) 4, 16alpha, 17beta-Estriol 3-(beta-Dglucuronide), (10) Cepharanthine, (11) Carapin-8 (9)-Ene, (12) 1-dodecanoyl-sn-glycerol.

Entry	1	2	3	4	5	6	7	8	9	10	11	12
**Druglikeness/Medicinal Chemistry**												
**Consensus Log *P*_o/w_**	4.52	−0.81	5.19	3.40	2.78	1.93	2.27	3.19	2.43	5.36	3.27	3.22
**Lipinski’s Rule**	Yes	No	Yes	Yes	Yes	Yes	Yes	Yes	Yes	Yes	Yes	Yes
**Bioavailability Score**	0.85	0.17	0.55	0.55	0.55	0.55	0.55	0.55	0.55	0.55	0.55	0.55
**PAINS (alerts)**	0	0	0	0	0	0	0	0	0	0	0	0
**Synthetic accessibility**	4.01	6.33	4.74	4.08	6.17	5.96	3.20	4.58	3.74	7.01	6.36	3.42
**Pharmacokinetics**												
**GI absorption**	High	Low	High	High	High	High	High	High	High	High	High	High
**BBB permeant**	Yes	No	No	No	No	No	No	No	Yes	No	No	Yes
**P-gp substrate**	No	Yes	No	No	No	No	No	No	Yes	No	Yes	No
**CYP1A2 inhibitor**	Yes	No	No	No	No	No	Yes	No	No	No	No	No
**CYP2C19 inhibitor**	No	No	Yes	No	No	No	No	Yes	No	No	No	No
**CYP2C9 inhibitor**	Yes	No	Yes	No	No	No	Yes	Yes	No	No	No	No
**CYP2D6 inhibitor**	Yes	No	No	No	Yes	No	Yes	No	Yes	No	No	Yes
**CYP3A4 inhibitor**	No	No	No	No	Yes	No	Yes	Yes	No	No	No	No
**Log Kp (cm/s)**	−4.31	−9.94	−5.2	−8.87	−7.63	−8.02	−0.55	−6.38	−6.32	−5.36	−7.53	−5.02

## Data Availability

All data of the current study are included in this article.
